# Genetic impacts on nigral iron deposition in Parkinson's disease: A preliminary quantitative susceptibility mapping study

**DOI:** 10.1111/cns.14135

**Published:** 2023-02-27

**Authors:** Jingjing Wu, Tao Guo, Cheng Zhou, Xueqin Bai, Xiaocao Liu, Luyan Gu, Min Xuan, Quanquan Gu, Peiyu Huang, Zhe Song, Baorong Zhang, Xiaojun Xu, Minming Zhang, Xiaojun Guan

**Affiliations:** ^1^ Department of Radiology, The Second Affiliated Hospital Zhejiang University School of Medicine Hangzhou China; ^2^ Department of Neurology, The Second Affiliated Hospital Zhejiang University School of Medicine Hangzhou China

**Keywords:** imaging genetic association, Parkinson's disease, quantitative susceptibility mapping, single nucleotide polymorphism, substantia nigra

## Abstract

**Background:**

Dysfunction of iron metabolism, especially in substantia nigra (SN), is widely acknowledged in Parkinson's disease (PD), but the genetic influence on iron deposition remains largely unknown. Thus, in this study, we aimed to investigate potential genetic impacts on iron deposition in PD.

**Methods:**

Seventy‐four subjects, including 38 patients with PD and 36 age‐matched normal controls, participated in this study. Imaging genetic association analysis was used to identify the specific influence of single nucleotide polymorphism (SNP) on iron‐related quantitative traits (QT). Genetic effects on iron deposition at the disease level, SNP level, and their interactive effect were highlighted.

**Results:**

Four strong SNP–QT associations were detected: rs602201‐susceptibility of bilateral SN, rs198440‐susceptibility of left SN, and rs7895403‐susceptibility of left caudate head. Detailed analyses showed that: (1) significant iron deposition was exclusively found in bilateral SN in PD; (2) altered polymorphisms of the A allele/A− genotype of rs602201 and G allele/G− genotype of rs198440 and rs7895403 were more frequently observed in PD; (3) for rs602201, among all subjects, A− genotype carriers showed significantly increased iron content than TT genotype in bilateral SN; for rs198440 and rs7895403, G− carriers showed increased iron content than AA genotype in left SN and left caudate head, respectively; and (4) rs602201 exhibited significant SNP‐by‐disease interaction in bilateral SN.

**Conclusions:**

This study shows that rs602201 and rs198440 have a stimulative impact on nigral iron deposition in PD, which provides improved understanding of iron‐related pathogenesis in PD, and specifically, that vulnerability to iron deposition in SN is genetic‐based.

## Graphical abstract

The dysfunction of iron metabolism, especially in substantia nigra (SN), in Parkinson's disease (PD) has been widely acknowledged, but the genetic influence on iron deposition remains largely unknown. Through the exploration of the relationship between genetics and iron metabolism in SN, this study discovers rs602201 and rs198440 have a stimulative impact on nigral iron deposition in PD.

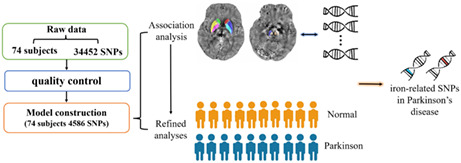

## 1 INTRODUCTION

Parkinson's disease (PD) is one of the most common neurodegenerative disorders, and is widely acknowledged as a disease with complex environmental and genetic interactions.^1^, ^2^ Although the etiology of PD remains unclear, it is well recognized that genetic factors play an important role in disease development. Mutations in many pathogenic genes are associated with both familial and sporadic PD.^2^ Excessive iron deposition in PD brain, and the related oxidative damage and Lewy body aggregation (which are closely associated with irreversible neurodegeneration), are the main pathological features of PD.^3^, ^4^, ^5^, ^6^ The underlying mechanisms of this abnormal iron metabolism remain largely unknown, therefore methods that combine genetic analysis along with measurement of brain iron may help understand the iron‐related pathogenesis of PD.

Measuring iron using magnetic resonance imaging (MRI) makes it possible to study iron‐related pathogenesis in PD in vivo. A newly developed MRI technology, quantitative susceptibility mapping (QSM), overcomes the non‐locality of tissue susceptibility in the brain, leading to a more precise measurement of brain iron with little effects of regional field inhomogeneity.^7^, ^8^ In accordance with histopathological findings,^9^ QSM has shown excessive iron deposition in subcortical nuclei of patients with PD, especially in the substantia nigra (SN).^10^, ^11^, ^12^, ^13^, ^14^ This suggests that QSM is a useful approach for capturing iron metabolism in vivo, and suggests that the SN is a core pathological target of iron dysfunction in PD. So far, only one study has explored the relationship between cortical iron deposition and gene expression (using the Allen Human Brain Atlas [AHBA]) in PD.^15^ The transcriptomic data of AHBA was created from the brains of only six donors, none of whom were used to obtain the imaging data of their study. In contrast, our research uses genetic data of participants combined with their own brain images, and is therefore closer to the “real” genetic background. Investigation of the genetic basis of iron metabolism in subcortical regions could lead to improved understanding of the iron‐related pathogenesis in PD.

Imaging genetics is a transdisciplinary field that examines associations between genetic variations, such as single nucleotide polymorphisms (SNPs), and imaging phenotypes (also known as quantitative traits (QT)). Association studies combining neurobiological imaging are closer to the underlying neurobiology of the disease, and are therefore more likely to identify disease‐related genes.^16^ Nevertheless, the majority of previous research has focused on the genetic effect of specific genes on brain alterations in PD,^17^, ^18^, ^19^ which are strongly dependent on prior knowledge and to some extent is biased. Thus, an imaging genetic association framework based on unscreened SNP data provides a novel approach for identifying disease‐relevant SNPs. In combination with iron‐related quantitative neuroimaging data, this approach can aid investigation of the genetic impacts on brain iron, which may subsequently facilitate the understanding of iron‐related pathogenesis in PD.

In the present study, we performed a preliminary examination of the associations between genes and iron deposition to determine the influence of genetics on iron‐related neurobiological traits in patients with PD. This approach can delineate the mechanism of nigral biased iron deposition from a genetic viewpoint. Furthermore, detailed analyses of significant SNPs with corresponding QTs were further examined to illustrate the genetic effects on iron deposition at the disease level, SNP level, and their interactive effect.

## 2 MATERIALS AND METHODS

### 2.1 Subjects

This study included 74 subjects: 38 patients with PD and 36 age‐matched normal controls (NC). All subjects were unrelated individuals. A diagnosis of PD was made by an experienced neurologist based upon the UK Parkinson's Disease Society Brain Bank criteria.^20^ All subjects were right‐handed and signed informed consent forms in accordance with approval of the Medical Ethics Committee of the hospital. Enhanced gradient echo T2 star‐weighted angiography (ESWAN) scanning, DNA detection, and basic demographic information (such as age, sex, and education) were obtained from all subjects. Disease duration, age at onset, Hoehn–Yahr scale (H‐Y), and Unified Parkinson's Disease Rating Scale (UPDRS) motor score were recorded from PD patients. Subjects with a history of other psychiatric or neurological disorders, brain trauma, or general exclusion criteria for MRI scanning were excluded from this study. For PD patients taking anti‐parkinsonian drugs, all examinations were performed after the withdrawal of all anti‐parkinsonian medicine overnight (at least 12 h) to ensure they were in “OFF” status.

### 2.2 MRI data acquisition

Subjects were scanned on a 3.0 Tesla MRI machine (Discovery 750; GE HealthCare) equipped with an eight‐channel head coil. During MRI scanning, the subjects' heads were fixed with foam pads, and earplugs were provided to reduce noise.

ESWAN images were acquired using a Gradient Recalled Echo sequence: repetition time = 33.7 ms; 1st echo time/spacing/8th echo time = 4.556 ms/3.648 ms/30.092 ms; flip angle = 20°; field of view = 240 × 240 mm^2^; matrix = 416 × 384; slice thickness = 2 mm; slice gap = 0 mm; 64 continuous axial slices.

### 2.3 MRI processing

QSM images were produced using the Susceptibility Tensor Imaging (STI) Suite V3.0 software package (https://people.eecs.berkeley.edu/~chunlei.liu/software.html) as follows: (1) the raw phase was unwrapped using Laplacian‐based phase unwrapping, and the normalized phase was calculated;^21^, ^22^ (2) the normalized background phase was removed using spherical‐mean‐value filtering (V_SHARP);^8^ and (3) tissue susceptibility was calculated using the STAR‐QSM method (streaking artifact reduction for QSM).^23^, ^24^ Mean magnetic susceptibility of each individual brain was used as the susceptibility reference. It is worth noting that selection of the susceptibility reference (e.g., cerebrospinal fluid^25^) did not influence the magnetic susceptibility measurement.

**FIGURE 1:**
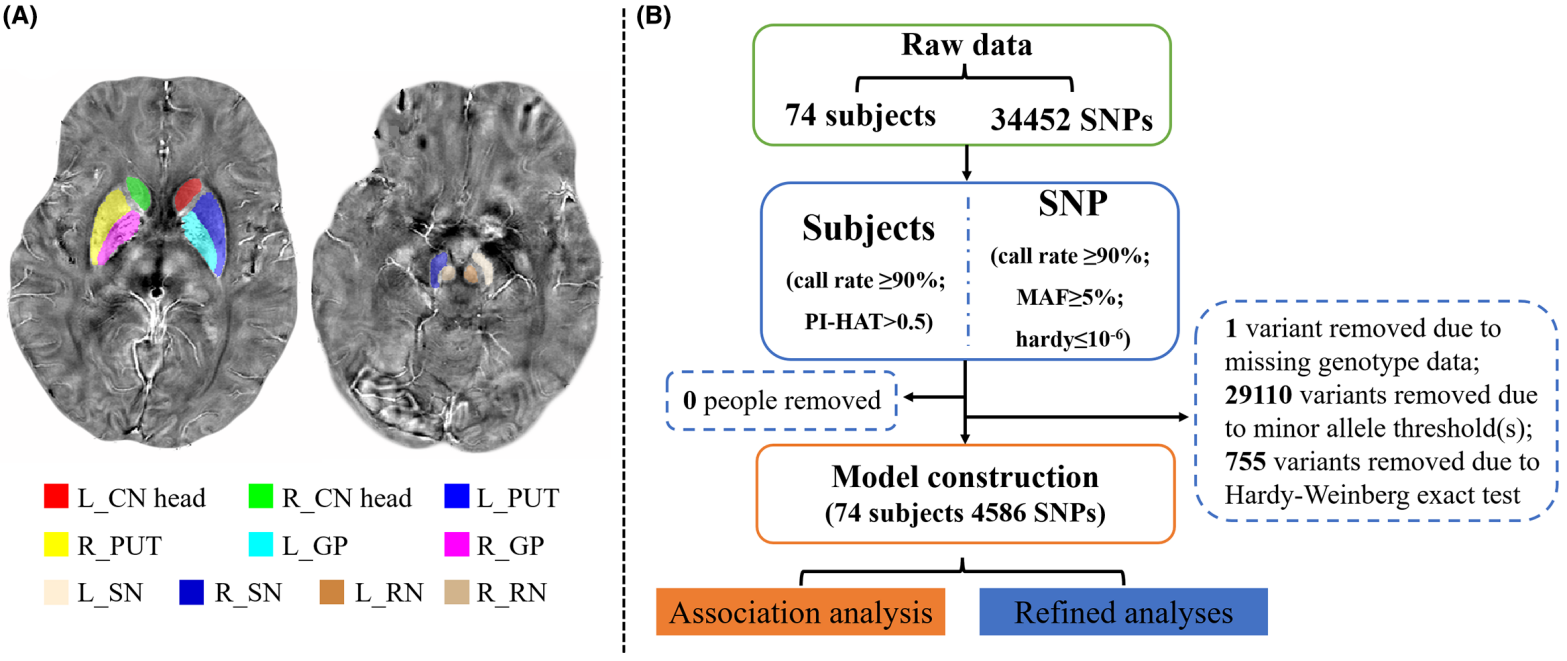
Data processing procedures. (A) The labels of subcortical nuclei in processed QSM images. (B) The framework of QC steps.

Tissue susceptibility of native subcortical nuclei in the basal ganglia and midbrain were extracted using the semi‐automatic segmentation method on the Advanced Normalization Tools (ANTs) as follows: (1) the native QSM image was registered to a newly constructed QSM template derived from a cohort of aging brains using ANTs‐SyN co‐registration algorithms;^26^, ^27^ (2) the labels covering subcortical nuclei were defined in the QSM template, including bilateral caudate head (CN head), putamen (PUT), globus palliduss (GP), SN, and red nucleus (RN; Figure 1A); (3) the labels in the QSM template were then warped to the native QSM image by inversing the transformation matrix calculated in the first step; and (4) visual examination and manual refinement were performed to ensure segmentation precision. Next, the susceptibility of each label in the native space was extracted and used as the imaging phenotype. Altogether, 10 QTs were obtained (Table 1).

**TABLE 1:** Imaging phenotypes.

Phenotype ID	Phenotype description
L_CN head	Magnetic susceptibility of left caudate head
R_CN head	Magnetic susceptibility of right caudate head
L_PUT	Magnetic susceptibility of left putamen
R_PUT	Magnetic susceptibility of right putamen
L_GP	Magnetic susceptibility of left globus pallidus
R_GP	Magnetic susceptibility of right globus pallidus
L_SN	Magnetic susceptibility of left substantia nigra
R_SN	Magnetic susceptibility of right substantia nigra
L_RN	Magnetic susceptibility of left red nucleus
R_RN	Magnetic susceptibility of right red nucleus

### 2.4 Genotyping

Genomic DNA of all subjects was extracted from peripheral blood samples using standard procedures and examined using an Illumina analyzer. Polymerase chain reaction and direct sequencing were used to sequence genes related to PD or parkinsonism symptoms (159 genes in total; Table 2).

**TABLE 2:** Genotyping panel.

Genes related to PD or parkinsonism symptoms (totally 159 genes)
ADAR, ADCY5, ADORA1, AIRE, ANG, ANO3, APTX, ATM, ATP13A2, ATP1A3, ATP6AP2, ATP7A, ATP7B, AXIN1, BRAF, C19orf12, CA2, CHCHD10, CHCHD2, CHMP2B, CIZ1, CNR1, CNR2, COASY, COL4A1, COL6A3, COQ2, CP, CTC1, CYP27A1, DAGLA, DAGLB, DCAF17, DCTN1, DDRGK1, DNAJC12, DNAJC13, DNAJC6, DRD1, DRD2, DRD3, DRD5, DST, ECHS1, ECM1, EIF4EBP1, EIF4G1, ERCC6, ERCC8, FA2H, FBXO7, FBXW7, FMR1, FOLR1, FTL, FUS, GALC, GATA3, GBA, GCDH, GCH1, GFAP, GIGYF2, GJD2, GLUD2, GNAL, GRN, HAPLN4, HPCA, HS1BP3, HTRA2, IFIH1, IL1B, INPP5F, ISG15, KCNS2, KMT2B, LINGO1, LRRK2, MAPT, MCCC1, MTHFR, NALCN, NDUFS4, NDUFV2, NKX2‐1, NPC1, NPC2, NR4A2, NUS1, OPTN, PACRG, PANK2, PARK7, PDE8B, PDGFB, PDGFRB, PINK1, PLA2G6, PNKD, PODXL, POLG, PRKN, PRKRA, PRRT2, PSEN1, PSEN2, PTRHD1, RAB29, RAB39B, RIC3, RNASEH2A, RNASEH2B, RNASEH2C, SAMHD1, SERAC1, SERPINA3, SGCE, SIGMAR1, SIPA1L2, SLC19A3, SLC1A2, SLC1A4, SLC20A2, SLC2A1, SLC30A10, SLC6A3, SMPD1, SNCA, SNCAIP, SORT1, SPR, SQSTM1, STK32B, SYNJ1, TAF1, TARDBP, TBCE, TBK1, TENM4, TH, THAP1, TIMM8A, TMEM230, TOR1A, TOR1B, TREM2, TREX1, TUBB4A, TYROBP, UBQLN2, UCHL1, USP46, VCP, VPS13C, VPS35, WDR45, XPR1, and ZFYVE26

### 2.5 Association analysis

From parkinsonism‐related genes, 34,452 SNPs were acquired. Quality control (QC) and association analyses were conducted using the PLINK software package v1.90 (http://www.cog‐genomics.org/plink2/). SNPs were excluded from this analysis that did not satisfy the following quality criteria: (1) call rate per SNP ≥90% (1 variant was excluded); (2) minor allele frequency ≥5% (29,110 variants were excluded); and (3) Hardy–Weinberg equilibrium test of *p* ≤ 10^−6^ (755 variants were excluded). Moreover, SNP QC was conducted among NC subjects only. As for subject level monitoring, the criteria were as follows: (1) call rate per subject ≥90% (no subject was excluded); and (2) identity check with PI‐HAT >0.5, which is a measure of subject relatedness (no subject was excluded). As a result, 4586 SNPs and all subjects remained for further analyses (Figure 1B). An association model was constructed to separately explore the main effects of all SNPs on each quantitative imaging phenotype. In each model, all 4586 SNPs were used as independent variables and the quantitative phenotype was regarded as the dependent variable, with the influence of age, sex, and education regressed out. The association results were thresholded at *p* < 0.05/4586/10 ≈ 10^−6^ (Bonferroni correction). A relatively less stringent threshold (*p* < 0.05/4586 ≈ 10^−5^, corrected at the SNP level) was used to identify additional SNP and imaging QT associations, as well as guide the detailed analyses (see below). A heat map was used to visualize the association results.

### 2.6 Detailed analyses

To comprehensively understand the genetic effects on iron deposition in PD, in‐depth analyses were performed. These focused on the association of significant SNPs with their corresponding QTs (revealed by the above association analysis). The detailed analyses included three steps: (1) allele/genotype frequency distribution calculation; (2) main effect of disease status and main effect of SNP estimation; and (3) SNP‐by‐disease interaction exploration.

### 2.7 Statistical analyses

Statistical analyses were performed using Statistical Product and Service Solutions (SPSS version 25.0). Demographics were obtained of subjects that passed the genotype QC procedure. Normal distribution of data was checked using the Kolmogorov–Smirnov test. Differences of age, sex, and education between groups were analyzed as appropriate using two‐sample *t*‐test, Pearson chi‐square test, or nonparametric test. *p* < 0.05 was regarded as statistically significant.

For the detailed analyses, the chi‐square test was used to calculate genetic distribution differences between groups at the allele and genotype level. The main effect of disease status and main effect of SNP on susceptibility of subcortical regions were analyzed using the general linear model, with the influence of age, sex, and education regressed out. Bonferroni correction was performed for multi‐comparisons. Furthermore, the SNP‐by‐disease interaction was explored, with *p* < 0.05 regarded as statistically significant.

## 3 RESULTS

### 3.1 Demographic characteristics

After QC of the genotyping data, 4586 SNPs and all subjects were included in the analysis. Table 3 shows the demographic characteristics. No significant differences were found in age between PD and NC (*p* = 0.096), whereas sex and education were statistically different (*p* = 0.005 and *p* = 0.010, respectively). Sex, education, and age were included as covariates in further analyses.

**TABLE 3:** Demographic characteristics.

Clinical variables	NC	PD	*p*‐value
Num	36	38	–
Age (years; mean ± SD)	60.56 ± 7.19	57.31 ± 9.21	0.096
Sex (F/M)	21/15	10/28	0.005*
Education (years; mean ± SD)	11.49 ± 3.69	9.03 ± 4.24	0.010*
Handedness (R/L)	36/0	38/0	–
Disease duration (years; mean ± SD)	–	2.32 ± 1.96	–
Age at onset (years; mean ± SD)	–	54.99 ± 9.22	–
H‐Y (median, range)	–	2.5 (1–3)	–
UPDRS motor score (mean ± SD)	–	19.13 ± 12.56	–

* Denotes the significant result.

### 3.2 Association analysis of imaging phenotypes

**FIGURE 2:**
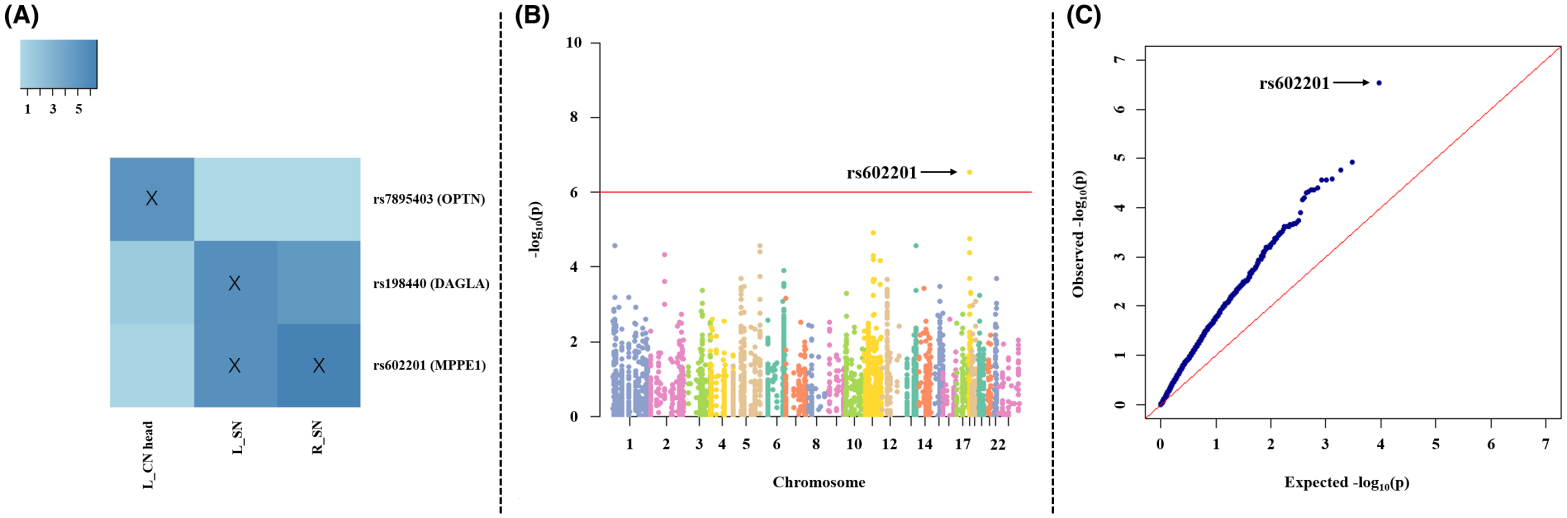
The association between genetic variations and imaging phenotypes. (A) Heat map with significant associations between SNP and QT at *p* < 10^−5^ (blocks labeled with “X”). Color key on the top left coded the magnitude of −log_10_(*p*‐values). (B, C) Manhattan and Q–Q plot of the most significant association (*p* < 10^−6^) (rs602201‐R_SN). Shown on (B) is the Manhattan plot of the −log_10_(observed *p*‐value). The horizontal line displayed the cutoff for *p* < 10^−6^. Shown on (C) is the Q–Q plot of the distribution of the observed *p*‐values (−log_10_(observed *p*‐value)) versus the expected *p*‐values (−log_10_(expected *p*‐value)) under the null hypothesis of no association.

All imaging genetic associations under a significance threshold of *p* < 10^−5^ are shown in Figure 2A. For convenience, each SNP is described using its ‘rs’ number together with its respective gene. Four strong SNP–QT associations were identified (see blocks labeled with “X” in Figure 2A): rs602201 (metallophosphoesterase, *MPPE1*) with susceptibility of left SN, rs602201 (*MPPE1*) with susceptibility of right SN, rs198440 (diacylglycerol lipase alpha, *DAGLA*) with susceptibility of left SN, and rs7895403 (optineurin, *OPTN*) with susceptibility of left CN head. Specifically, the association between *MPPE1* SNP rs602201 and magnetic susceptibility of right SN showed the most significant result with *p* < 10^−6^. For further examination, a Manhattan plot and quantile–quantile (Q–Q) plot were used to visualize the most significant association between a SNP and its corresponding imaging phenotype, that is, rs602201‐R_SN (Figure 2B,C). In the Q–Q plot, the *p*‐value in the upper tail of the distribution shows a significant deviation, suggesting there is a significant association between the QT and SNP.

### 3.3 Detailed analyses

Additional detailed analyses were used to further illustrate the importance of identifying imaging genetic associations for PD. First, we calculated genetic distribution differences between PD and NC at the allele and genotype level for rs602201, rs198440, and rs7895403. The results are shown in Table 4. Compared with NC, the frequency of the altered A allele and A− genotype (AT + AA genotype) for rs602201 were significantly higher in PD patients (both *p* < 0.001). Similar results were obtained for the altered G allele and G− genotype (GA + GG genotype) for rs198440 (both *p* < 0.001), and rs7895403 (*p* = 0.002 and *p* = 0.020, respectively).

**TABLE 4:** Frequency distribution at allele and genotype level.

Levels	NC	PD	*p*‐value
*rs602201 (MPPE1)*			
Allele (T/A)	64/8	49/27	<0.001*
Genotype (TT/A−)	29/7	15/23	<0.001*
*rs198440 (DAGLA)*			
Allele (A/G)	64/8	36/40	<0.001*
Genotype (AA/G−)	29/7	11/27	<0.001*
*rs7895403 (OPTN)*			
Allele (A/G)	63/9	50/26	0.002*
Genotype (AA/G−)	29/7	21/17	0.020*

*Note*: A−, AT + AA genotype; G−, GA + GG genotype.

* Denotes the significant result.

**FIGURE 3:**
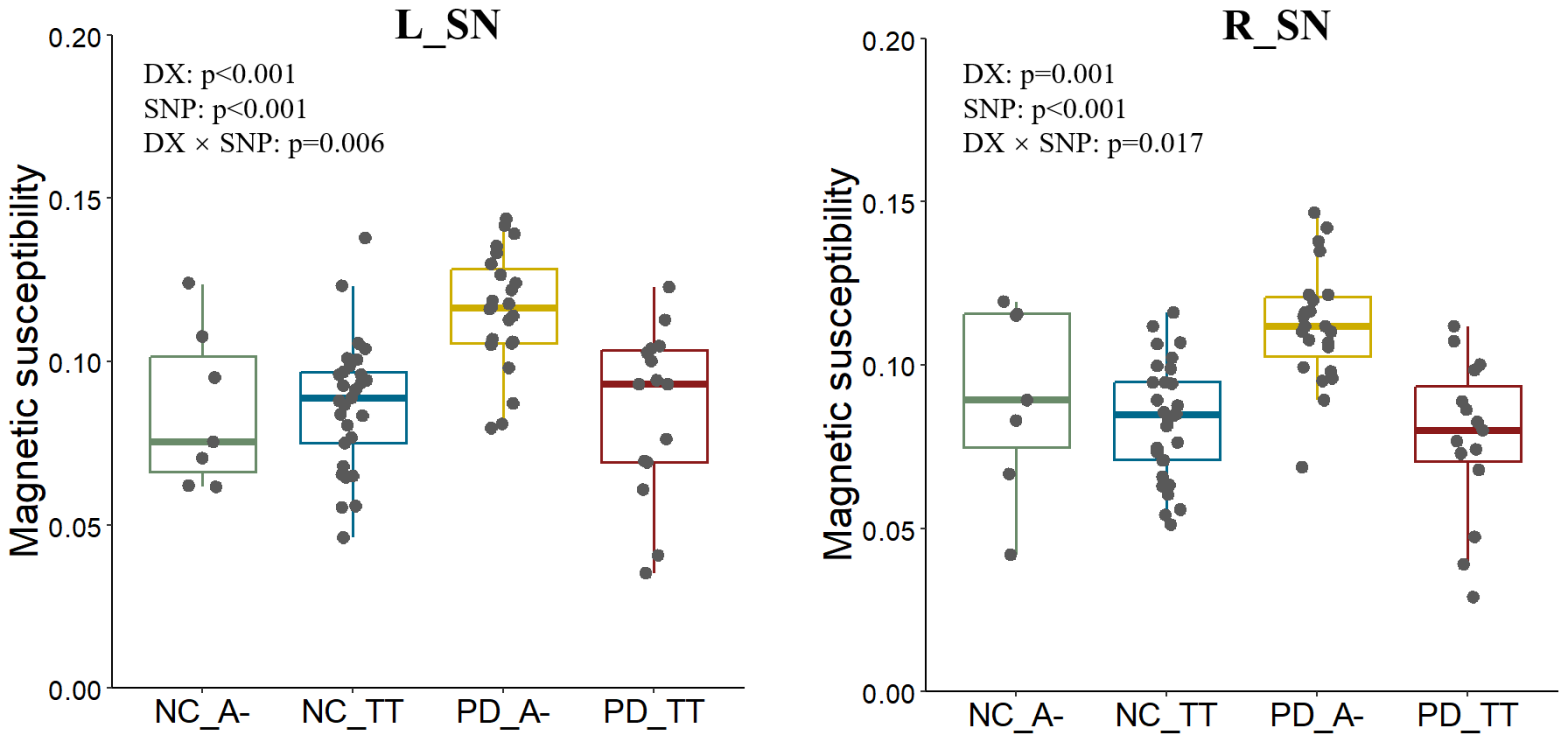
The main effect of diagnosis, SNP, and their interactions of rs602201 in bilateral SNs. DX, the main effect of diagnosis; SNP, the main effect of SNP (includes all subjects); DX × diagnosis, the interaction of diagnosis and SNP; A−, AT + AA genotype.

The main effect of disease status, the SNP, and their interactive effect on the corresponding QT were further explored. Compared with NC, patients with PD showed increased iron content in bilateral SN (*p* < 0.001 for left SN and *p* = 0.001 for right SN; Figure 3), whereas the iron content of left CN head did not show any intergroup difference (*p* = 0.426). After evaluating the difference of magnetic susceptibility between groups of different disease status, we next estimated the SNP main effect. For rs602201, among all subjects, A− genotype carriers showed significantly increased magnetic susceptibility in bilateral SN (both *p* < 0.001) compared with TT genotype subjects. This indicates a higher iron content in bilateral SN in A− carriers (Figure 3). To determine whether a particular disease group was responsible for this effect, we examined its effect within each group (i.e., PD group and NC group). The significance pattern for iron deposition (A− > TT) in bilateral SN only existed in patients with PD (*p* < 0.001 for left SN and *p* < 0.001 for right SN), which suggests a possible SNP‐by‐disease interactive effect for rs602201 on nigral iron deposition. In subsequent analysis, as we speculated, a SNP‐by‐disease interactive effect was associated with bilateral nigral iron deposition (*p* = 0.006 for left SN and *p* = 0.017 for right SN; Figure 3). This suggests that PD patients with a rs602201 A− genotype are particularly vulnerable to iron deposition in bilateral SN.

Similar to previous analysis, when including all subjects, G− carriers showed increased iron content compared with the AA genotype for rs198440 on left SN and rs7895403 on left CN head (both *p* < 0.001). Focusing on the genetic effect within each group, we found that for rs198440, a higher iron content of G− > AA was only found among patients with PD (*p* = 0.034) and not in NC subjects (*p* = 0.199). For rs7895403, both PD and NC groups showed a higher iron content in G− carriers (*p* = 0.005 and *p* = 0.040, respectively). However, both SNPs showed no SNP‐by‐disease interactive effect (rs198440‐by‐L_SN, *p* = 0.520; rs7895403‐by‐L_CN head, *p* = 0.578).

## 4 DISCUSSION

Using imaging genetic association analysis, we find some preliminary findings: (1) two variants, rs602201 (*MPPE1*) and rs198440 (*DAGLA*), were associated with nigral iron deposition; and altered genotype carriers have significantly increased iron content compared with wild‐type genotype subjects; (2) the altered allele and genotype for both rs602201 and rs198440 were more frequently observed in PD patients; (3) for both rs602201 and rs198440, only PD patients have the pattern that carriers with altered polymorphisms exhibit increased iron content compared with wild‐type genotype subjects; furthermore, rs602201 exhibited a significant SNP‐by‐disease interaction in bilateral SN, indicating that PD patients with the rs602201 A− genotype may be particularly vulnerable to iron deposition in bilateral SN; and (4) the variant rs7895403 (*OPTN*) was associated with iron deposition in the left CN, but no intergroup difference of iron deposition within this nucleus was observed.

### 4.1 Stimulative impact of rs602201 ( *MPPE1* ) and rs198440 ( *DAGLA* ) on nigral iron deposition in PD

Two variants, rs602201 (*MPPE1*) and rs198440 (*DAGLA*), were associated with nigral iron deposition in PD, with the altered genotype carriers showing significantly increased iron content compared with wild‐type genotype subjects at the whole subject range. This suggests a close relationship between these two SNPs and nigral iron deposition. Furthermore, altered polymorphisms of the A allele/A− genotype for rs602201 and G allele/G− genotype for rs198440 were more frequently observed in PD, indicating that these two SNPs might exert a pathological contribution to nigral iron metabolism in PD. The *MPPE1* gene encodes a protein that is a member of the calcineurin‐like phosphoesterase superfamily.^28^ Phosphoesterase is involved in a variety of phosphorylation–dephosphorylation processes. Emerging data suggest that alterations in protein phosphorylation play a role in dopaminergic neurotransmission,^28^ for example, dopaminergic transporter activity is regulated by a phosphorylation‐mediated process.^29^ Thus, disturbance of *MPPE1* could impair physiological regulation of the dopaminergic system, which to some extent may give rise to PD development. Besides, MPPE1 protein is a metal‐dependent phosphoesterase containing metal binding and active sites, including iron binding sites, that is widely expressed in the brain, especially the SN.^28^, ^30^, ^31^ This suggests that the *MPPE1* gene may exert a pivotal influence on nigral iron deposition in PD. As for the *DAGLA* gene, it encodes a protein that is a diacylglycerol lipase, which is a synthesis enzyme of the endocannabinoid system (ECS).^32^ The cannabinoid 1 receptor is one of the major receptors in the ECS and is highly expressed in the SN,^31^ the signaling pathway of which interacts with dopaminergic D1‐/D2‐like receptors.^33^, ^34^, ^35^ This suggests that the ECS is related to the dopaminergic system and might play an important role in the pathogenesis of PD. In addition, cannabis can relieve the symptoms of PD via its antioxidant properties, suggesting that excessive oxidative stress may be caused by dysfunction of the ECS in patients with PD.^33^ Intriguingly, excessive regional iron deposition can reinforce the oxidative stress effect, causing neuronal dysfunction and death.^3^, ^36^ Together with our findings, dysfunction of the ECS may interact with nigral iron deposition and combine to induce excessive oxidative stress, leading to neuronal death in PD. From this, we suggest that genetic variation of *DAGLA* via an iron‐interactive ECS is associated with PD pathogenesis. Therefore, our findings indicate that these two SNPs have a pathological contribution to nigral iron metabolism, which may exert a hazardous effect on PD pathology.

Focusing on the genetic effect within each group, only patients with PD showed increased iron content in altered polymorphism carriers compared with wild‐type genotype subjects for both rs602201 and rs198440. This indicates that these two SNPs have specific stimulative effects on nigral iron deposition in PD. However, in a subsequent analysis, a SNP‐by‐disease interactive effect was only observed for rs602201, reinforcing the notion that rs602201 has a pathogenic effect on nigral iron deposition in PD. Therefore, we speculate that PD patients with the rs602201 A− genotype may be particularly vulnerable to nigral iron deposition. The SNP rs602201 (*MPPE1*) may be a plausible biological candidate trait for nigral iron deposition. Thus, its altered polymorphism may leave carriers susceptible to PD pathology. We did not find a SNP‐by‐disease interaction for rs198440, which may suggest that the effect of PD heterogeneity is unavoidable for detecting association between SNP rs198440 and nigral iron accumulation. Therefore, these findings not only reveal the important role of SNP rs602201 in specifically modulating pathogenic iron deposition in SN in PD, but suggest the genetic effect of certain SNPs (e.g., rs198440) may be diverse from disease status.

### 4.2 General genetic effects of rs7895403 ( *OPTN* ) on left CN iron deposition in PD

The variant rs7895403 (*OPTN*) was associated with left CN iron deposition, and when all subjects were included, G− carriers showed increased iron content compared with the AA genotype. This indicates its genetic effect on iron deposition among the general population. OPTN protein is an autophagy receptor that is involved in mitophagy.^37^ Previous studies have reported that OPTN variation can influence iron metabolism by inhibiting the uptake of transferrin, resulting in reduced cellular transferrin receptor levels in the cell.^38^ Aberrant iron deposition can backwardly influence mitochondrial respiration and lead to mitophagy‐related dysfunction,^3^ demonstrating an interaction between OPTN protein and iron deposition.

However, we observed no intergroup difference of iron deposition in left CN, which is consistent with former studies showing unchanged iron content in left CN between PD and NC.^11^, ^12^ This may be due to the region‐specific iron deposition pattern in PD, as shown in our study, with the iron deposition in subcortical nuclei not distributed homogeneously, but instead preferentially deposited in SN.

Although the altered polymorphism G allele/G− genotype for rs7895403 was more frequently observed in PD, both NC and PD groups showed a higher iron content in altered polymorphism carriers compared with wild‐type genotype subjects within each particular group. This indicates that the genetic effect was not specific. Besides, a SNP‐by‐disease interaction was not detected for rs7895403, which strengthens the likelihood that rs7895403 has a genetic effect on iron deposition, but this effect is general and not specific for PD.

### 4.3 Limitations

We acknowledge potential limitations of our study. First, our sample size was relatively small, although previous research has shown that association studies combining QT have increased statistical power, which could decrease sample size requirements.^39^ Studies with larger samples combining multi‐center data or lengthening the data collection period are required in the future. Second, our study lacks a holistic perspective in regard a whole genome and/or whole brain scale, although we already selected the genes related to PD or parkinsonism symptoms. In the future, genome‐wide association studies should be used to remove the possibility of missing important associations.

## 5 CONCLUSIONS

This study discovered two variants, rs602201 (*MPPE1*) and rs198440 (*DAGLA*), that have a significant stimulative impact on nigral iron deposition in PD. However, only the rs602201 (*MPPE1*) polymorphism has a specific interactive effect on bilateral SN, indicating that PD patients with rs602201 polymorphism may be particularly vulnerable to nigral iron deposition. In contrast, the variant rs7895403 (*OPTN*) was only found to have a general genetic effect on iron deposition in CN among the whole population. Altogether, these findings provide a deeper understanding of iron‐related pathogenesis in PD, specifically that iron metabolism involves genetic interactions and vulnerability to iron deposition in the SN is genetic‐based.

## AUTHOR CONTRIBUTIONS

All of the coauthors listed meet the criteria for authorship. JJW was involved with data acquisition, study concept and design, data analysis and interpretation, manuscript drafting and revising. XJG, TG, and CZ were involved with data acquisition, data analysis and interpretation. XQB, XCL, LYG, MX, QQG, and PYH were involved with data acquisition. ZS and BRZ were involved with PD patients' recruitment. XJX was responsible for data interpretation, manuscript revision, funding obtaining and study supervision. XJG and MMZ was responsible for study concept, manuscript revision, funding obtaining and study supervision. All authors read and approved the final manuscript.

## CONFLICT OF INTEREST STATEMENT

The authors reported no biomedical financial interests or potential conflicts of interest.

## INFORMED CONSENT

Informed consent was obtained from all individual participants included in the study.

## Data Availability

The materials used and/or analyzed during the current study are available from the corresponding author on reasonable request.
